# Gamma Irradiation Effect on Polymeric Chains of Epoxy Adhesive

**DOI:** 10.3390/polym16091202

**Published:** 2024-04-25

**Authors:** Carino Ferrante, Leonardo Lucchesi, Alessia Cemmi, Ilaria Di Sarcina, Jessica Scifo, Adriano Verna, Andrea Taschin, Luca Senni, Marco Beghini, Bernardo Disma Monelli, Fabrizio Raffaelli

**Affiliations:** 1 CNR-SPIN, c/o Dip.to di Scienze Fisiche e Chimiche, Via Vetoio, 67010 L’Aquila, Italy; 2 FSN-FISS-SNI Laboratory, ENEA, Via Anguillarese, 301, 00123 Rome, Italy; 3 Istituto Nazionale di Fisica Nucleare (INFN), Sezione di Pisa, Largo Bruno Pontecorvo 3, 56127 Pisa, Italy; leonardo.lucchesi@pi.infn.it (L.L.); fabrizio.raffaelli@pi.infn.it (F.R.); 4 Dipartimento di Ingegneria Civile e Industriale, Università di Pisa, Largo Lucio Lazzarino 1, 56122 Pisa, Italy; marco.beghini@unipi.it (M.B.); bernardo.disma.monelli@unipi.it (B.D.M.); 5 ENEA–Nuclear Department, NUC-IRAD-GAM Laboratory, Via Anguillarese 301, 00123 Rome, Italy; alessia.cemmi@enea.it (A.C.); ilaria.disarcina@enea.it (I.D.S.); jessica.scifo@enea.it (J.S.); adriano.verna@enea.it (A.V.); 6 ENEA–Fusion Physics Division, Via E. Fermi, 45, 00044 Rome, Italy; andrea.taschin@enea.it (A.T.); luca.senni@cnr.it (L.S.); 7 Consiglio Nazionale delle Ricerche, Istituto Nazionale di Ottica, CNR-INO, Via Nello Carrara 1, 50019 Firenze, Italy; 8 CNR–Institute for Applied Mathematics ‘Mauro Picone’ (IAC), Via dei Taurini, 19, 00185 Rome, Italy

**Keywords:** epoxy adhesive, *γ*ray, polymeric chains

## Abstract

The study of materials for space exploration is one of the most interesting targets of international space agencies. An essential tool for realizing light junctions is epoxy adhesive (EA), which provides an elastic and robust material with a complex mesh of polymeric chains and crosslinks. In this work, a study of the structural and chemical modification of a commercial two-part flexible EA (3M™ Scotch-Weld™ EC-2216 B/A Gray), induced by ^60^Co gamma radiation, is presented. Combining different spectroscopic techniques, such as the spectroscopic Fourier transform infrared spectroscopy (FTIR), the THz time-domain spectroscopy (TDS), and the electron paramagnetic resonance (EPR), a characterization of the EA response in different regions of the electromagnetic spectrum is performed, providing valuable information about the structural and chemical properties of the polymers before and after irradiation. A simultaneous dissociation of polymeric chain and crosslinking formation is observed.The polymer is not subject to structural modification at an absorbed dose of 10 kGy, in which only transient free radicals are observed. Differently, between 100 and 500 kGy, a gradual chemical degradation of the samples is observed together with a broad and long-living EPR signal appearance. This study also provides a microscopic characterization of the material useful for the mechanism evaluation of system degradation.

## 1. Introduction

The material resistance to ionizing radiations is a challenging property, that enables the implementation in radiation-hardened or resilient systems, largely used in nuclear reactors, high-energy physics (HEP), and space applications. The long penetration depth of gamma rays (γrays), due to their high energy, prevents easy shielding for this kind of radiation. For this reason, irradiation tests with γray sources are indispensable for components used in an environment with ionizing radiation [1]. Owing to their lightness, resistance, and easy implementation, adhesive junctions based on polymers could represent a useful tool in these hard environments [2]. These systems are characterized by long polymeric chains and crosslinks (bonds between polymeric chains), whose density determines the stiffness and brittleness of polymers. The polymeric chains scission (i.e., skeletal bonds rupture) and the loss of crosslinking are the main culprits of polymeric softening [3]. The ionizing radiation induces in EA two different and opposite effects: the increase in crosslinking density and chemical bond breaking (with the generation of free radicals) [4,5].

**Figure 1:**
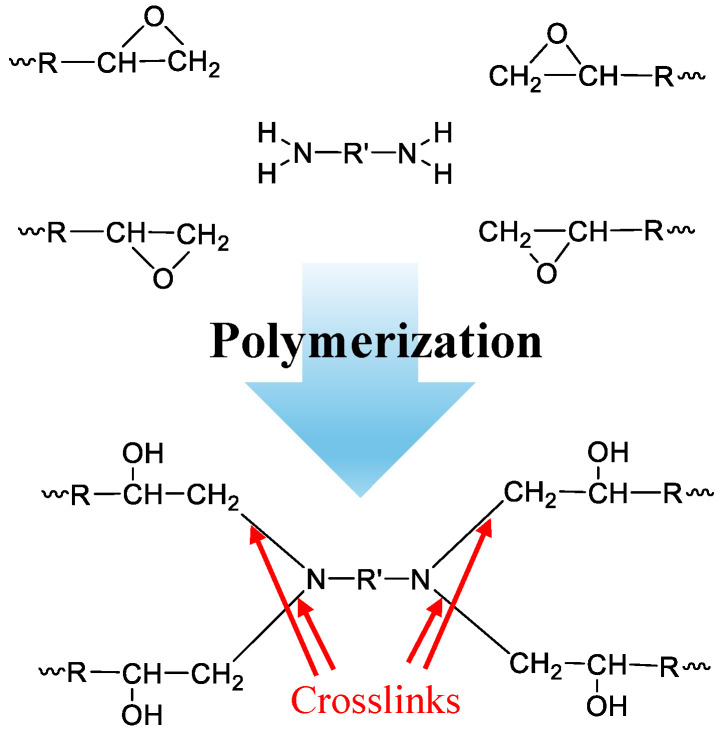
Sketch of typical reaction for the polymeric crosslinking [9,10]. Specifically, we report the bond formation between a diamine compound and 4 epoxy group of polymeric chain. Depending on how many amino groups there are in the crosslinker, the crosslink structure changes.

Epoxy adhesives are considered a very hard polymer, withstanding extreme environments for a considerable time. For instance, they are used as adhesives [6], matrices for composite materials [7], and encapsulating agents [8]. They are characterized by the presence of polymers or prepolymers that have epoxy functional groups. The crosslinking processes are based on the free electron available after the breaking of the epoxy ring, induced by the interaction with an appropriate hardener. One of the possible reaction pathways, reported as an example [9,10], is shown in Figure 1.

Several studies of the mechanical and structural properties of adhesives are performed for their implementation in environments with ionizing radiation [11,12,13,14,15,16,17].

In our work, the interest is focused on a commercial two-part EA, namely, 3M™ Scotch-Weld™ EC-2216 B/A Gray (3M2216). The mechanical properties of 3M2216 are studied [18,19] also after the gamma irradiation exposure test [20,21]. Here, the γray-induced modification of vibrational and free-radical properties of the polymer after irradiation tests are analyzed with three different techniques: Fourier-transform infrared spectroscopy (FTIR), electron paramagnetic resonance (EPR), and Terahertz time-domain spectroscopy (THz-TDS). The FTIR results highlight a gradual degradation of the polymeric chains for doses higher than 10 kGy, which do not affect THz measurements up to 90 cm^−1^. Moreover, the EPR measurements show the γray-induced formation of two types of free radicals. The first one has a recombination time scale of 70 h, whereas the latter one does not show an evident recombination and it is proportional to the γray-induced structural modification observed by FTIR measurements.

## 2. Materials and Methods

### 2.1. Sample Preparation

For the spectroscopic properties study, bulk specimens of EA were chosen. A custom mold was designed, composed of two thick aluminum plates glued to polytetrafluoroethylene (PTFE) layers, separated using a shaped PTFE mask of the same thickness as the specimens produced. Applying proper pressure on the mold with 18 studs during the first phases of the cure, bulk specimens were obtained with a thickness of 3.2 mm [20]. The same mold has been used to produce thinner samples of about 150 µm, but in this case, instead of a mask, properly calibrated spacers were used to achieve the desired thickness. The samples, handled without skin contact (to avoid superficial organic contamination detectable with FTIR), were sliced opportunely to fit our EPR spectroscopic systems.

### 2.2. Gamma Irradiation Test

Gamma irradiation tests were performed at the ^60^Co Calliope facility at ENEA Casaccia Research Center, Rome, Italy [22]. The Calliope facility is involved in radiation processing research and qualifications on materials and devices for hostile radiation environments, such as nuclear plants, space, and HEP experiments. Irradiation tests were carried out in air at the dose rate of 5.2 kGy_water_/h (equivalent dose in water) experimentally determined by Fricke absolute dosimetry (error of 2.5% [22]) at four absorbed dose values (10 kGy, 100 kGy, 300 kGy, and 500 kGy). The samples were exposed to air during the irradiation in order to simulate the worst oxidative condition.

### 2.3. FTIR Spectroscopy

**Figure 2:**
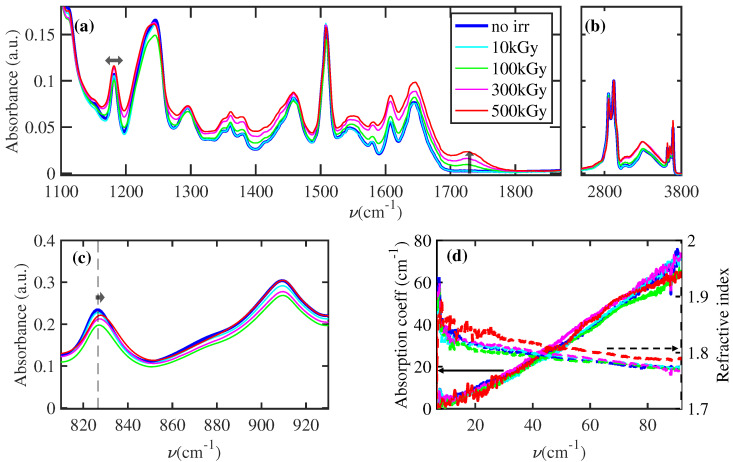
Epoxy adhesive FTIR and THz-TDS measurements at different total absorbed doses are shown. Each FTIR line is the average of more than 10 FTIR measurements normalized to the 2923 cm^−1^ peak. Different FTIR spectral regions of interest are highlighted in (**a**–**c**). The black arrows of ∼1180 cm^−1^, ∼1725 cm^−1^, and 827 cm^−1^ indicate the γray-induced broadening, intensity increasing, and frequency shift of FTIR peak, respectively. Differently, the refractive index and absorption coefficient obtained by the THz-TDS setup are reported in (**d**). Part of the figure was reproduced/adapted with permission from [20], Elsevier, 2021.

Vibrational spectroscopy is a powerful tool to explore the dynamic of chemical bonds in a material. Specifically, the modifications of intensity, bandwidth, and position of FTIR peaks provide unique information about the chemical and structural evolution of the system. FTIR measurements were performed in attenuated total reflection (ATR) mode before and after each irradiation step by using a SPECTRUM 100 Spectrophotometer (Perkin Elmer, Waltham, MA, USA) equipped with ZnSe crystal. The spectra were recorded in air with a spectral resolution of 4.7 cm^−1^ and step of 1 cm^−1^, in the range 800–3800 cm^−1^. The averaging processes are described in Figure 2.

### 2.4. EPR Spectroscopy

EPR spectra were acquired using a BRUKER EPR (BRUKER, Billerica, MA, USA) e-scan spectrometer operating in the X-band frequency (9.4 GHz) with a field modulation frequency of 86 kHz and modulation amplitude of 5.152 G. The EPR spectra were recorded, before and after the irradiation tests, at a central magnetic field of 3466 G with a sweep width of 160 G, microwave power of 0.14 mW, and microwave frequency of 9.75 GHz.

### 2.5. THz Time-Domain Spectroscopy

A THz time-domain spectroscopy (TDS) measurement system was employed to investigate the optical properties of the sample in the frequency range of 0.1 to 4 terahertz [23]. THz pulses are produced by exciting a photoconductive antenna (PA) by 100 fs infrared laser pulses generated by a T-light 780 nm fiber laser from MenloSystems (Martinsried, Germany).The emitter PA is biased with a sinusoidal tension of 0–30 V at the frequency of 10 KHz. THz pulses are then collimated and focused onto the sample under investigation using two off-axis parabolic mirrors. The transmitted THz pulses are collected by a second couple of off-axis parabolic mirrors and detected using a second PA gated by a twin laser pulse. Then, the receiver PA current is sent to a lock-in amplifier together with the modulation voltage reference. The temporal evolution of the THz electric field is traced by recording the output at changes in the time delay between the pump and probe pulses.

## 3. Results and Discussion

### 3.1. Structural Change of Polymeric Chains

Figure 2a–c show the average of FTIR measurements at different γray doses, normalized to the maximum absorbance value of the peak at 2923 cm^−1^ (Figure 2b), corresponding to the C-H stretching vibrational bands. The high intensity and the low chemical specificity of this latter band allow us to consider this peak as a good candidate for normalization. At each dose, between 10 and 12 samples with different aging conditions (between 48 and 150 days) were irradiated. The absence of FTIR modification induced by aging for this specific EA justifies our averaging process in Figure 2. Each sample was measured before and after irradiation. Consequently, the blue line is an average of FTIR measurements performed on 44 samples before irradiation. The data are completed by the results obtained in the THz-TDS region, reported in Figure 2d. In this latter frequency range, there does not appear any absorption peak and the measurements of absorption (solid lines) and real part of refractive index (dashed lines) are not affected by the gamma irradiation, highlighting a negligible modification of material crystallinity [24,25,26].

Differently, the absorption peak observed with the FTIR technique reveals a γray-induced modification of intensity, bandwidth, and position. These phenomena are interpreted below as an effect induced by chemical modification in the polymeric chains and crosslinks.

**Figure 3:**
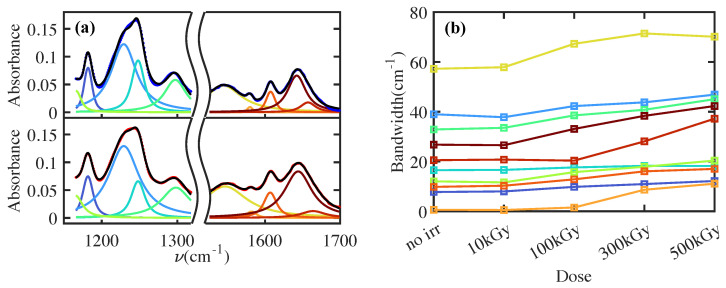
Fit of two FTIR spectral regions with a sum of Lorentzian convoluted with the spectral resolution of the instrument (Gaussian profile with variance 4.7 cm^−1^). (**a**) The best fit (black lines) of the nonirradiated FTIR spectrum (blue dots in the upper panel) and FTIR spectrum at a dose of 500 kGy (red dots in the lower panel) with the convoluted Lorentzian contribution with colored solid lines. The bandwidth of Lorentzian peaks is reported with the same colors in (**b**) for different doses. The profiles highlight a collective peak broadening related to a more heterogeneous chemical structure of polymeric chains and crosslinks.

In the fingerprint region (Figure 2a), a dose-dependent increase in the C=O peak at 1725 cm^−1^ is observed [20]. This latter indicates a fragmentation of carbon polymeric chains with a subsequent bonding of carbon atoms with oxygen. Similar conclusions can be obtained by observing the peak in Figure 2a at a lower frequency than the C=O peak. Specifically, a slight increase in intensity and a broadening of peaks is observed. To quantify the dose-dependent broadening, a fit of the spectral region is performed for all the doses and presented in Figure 3. The spectra are fitted by a sum of Lorentzian, convoluted with the spectral resolution of the instrument (Gaussian profile with variance 4.7 cm^−1^). The fitted profile of the nonirradiated (top) and of the irradiated at the maximum dose of 500 kGy (bottom) samples are shown in Figure 3a with the relative convoluted Lorentzian contributions. Figure 3b shows the dose dependence of the Lorentzian peak bandwidth (the colored lines refer to the Lorentzian peaks in Figure 3a with the same color). The observed collective broadening indicates an inhomogeneous process, related to a more complex combination of molecular bonds in the polymer [27]. This latter feature with the appearance of the C=O peak can be summarized by a blending of the chemical structure with a heavy oxidation of carbon atoms. In Figure 2c, a dose-dependent blueshift of 827 cm^−1^ is observed. This peak, fundamental for the characterization of epoxy resin [28,29], is assigned to an out-of-plane bending of the =C-H in the maleimide unit [30]. The polymerization process generates a blueshift of this peak [31]. Consequently, our shift can be ascribed to an increase in crosslinks in the polymers induced by γrays interaction.

**Figure 4:**
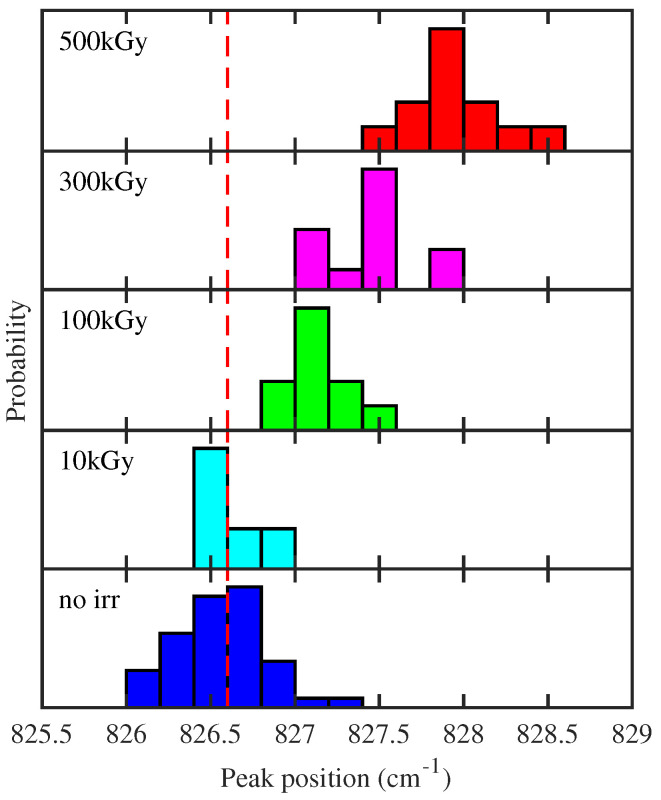
Distribution of the exact maximum position for the FTIR peak around 827 cm^−1^ for the different samples before irradiation (lowest panel) and after the irradiation at the different indicated doses (higher panels). The spectra reported in Figure 2 are, instead, the average of all the spectra acquired before the irradiation and after. The position is obtained by fitting with a Gaussian spectral range of 6 cm^−1^ around the peak maximum. The red dashed line is a visual guide to highlight the dose-dependent blueshift.

The histograms in Figure 4 show the distribution of the exact maximum position of the peak around 827 cm^−1^ for individual samples before and after the irradiation processes. They allow us to quantify the shift towards higher energy and its dependence on absorbed dose and to estimate the fluctuation of the position among samples with the same absorbed dose, which is lower than the blueshift for the highest explored dose.

Moreover, Figure 2b shows the increase in the broad peak between 3000 and 3500 cm^−1^, assigned to the O-H stretch. This evidence can be affected by two different effects: (i) the increase in absorbed water in the material; (ii) gamma irradiation can break chemical bonds within the epoxy resin, leading to the formation of free radicals which can react with oxygen to form oxidized products containing hydroxyl (-OH) groups.

**Figure 5:**
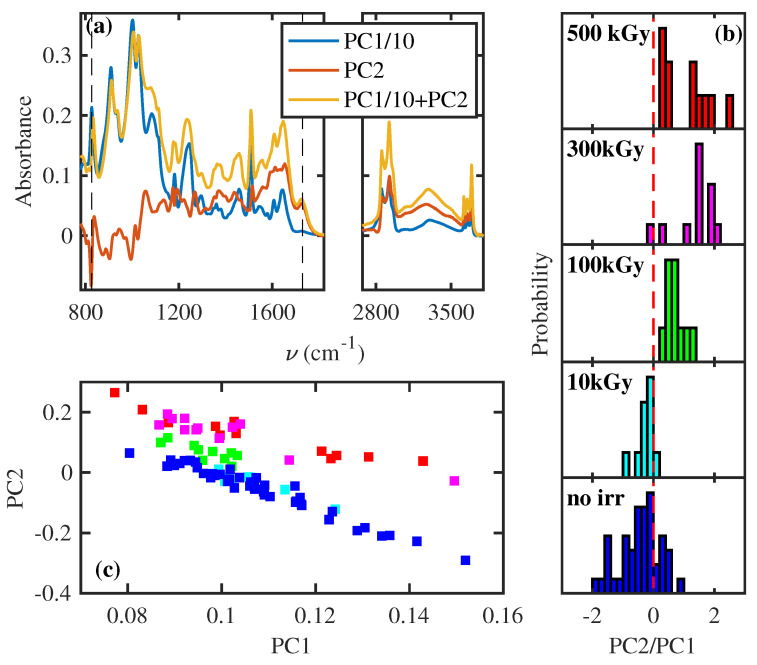
PCA of FTIR spectra with two components. (**a**) The spectra associated with the PC1 and PC2. The spectrum of PC1 is divided by a factor of 10 for graphical reasons. A combination of two spectra is also reported, with the yellow line discerning the role of PC2 addition in the spectral profile. (**b**) Histogram of the ratio of coefficient PC2 and PC1 for different doses. (**c**) The scatter plot of coefficient PC2 and PC1. The colors of bars and squares in (**b**,**c**) indicate the γray dose of the samples in agreement with Figure 2.

To summarize all these features, a principal component analysis (PCA) [32] with two components was performed on all FTIR spectra of each sample before and after the irradiation at different absorbed doses. The first principal component (PC1) represents the main peaks contribution observed in each spectrum (see the blue line in Figure 5a). On the contrary, the spectrum of the second principal component (PC2) represents the fluctuations of PC1 that allow us to reproduce the increase in the 1725 cm^−1^ peak, the broadening of peaks in the fingerprint, and the shift of 827 cm^−1^ peak, as testified by the yellow line of Figure 5a. Each FTIR spectrum collected is reproduced by a combination of coefficients PC1 and PC2, reported as a histogram (Figure 5b) and scatter plot (Figure 5c). Both figures show that the γray irradiation increases PC2 values. In particular, doses ≥ 100 kGy induce spectral modifications that are easily detectable with PCA algorithms or with a careful analysis of FTIR spectra.

Summing up, the analysis of FTIR spectra allows us to unveil the effect of γrays, which simultaneously perform the scission of the polymeric chain with the consequent reduction in the length (see Figure 2a and Figure 3) and increase the crosslinking (see Figure 2b and Figure 4). These effects are statistically relevant, as quantified through principal component analysis on single measurements (see Figure 5).

### 3.2. Dynamic of Free Radicals

**Figure 6:**
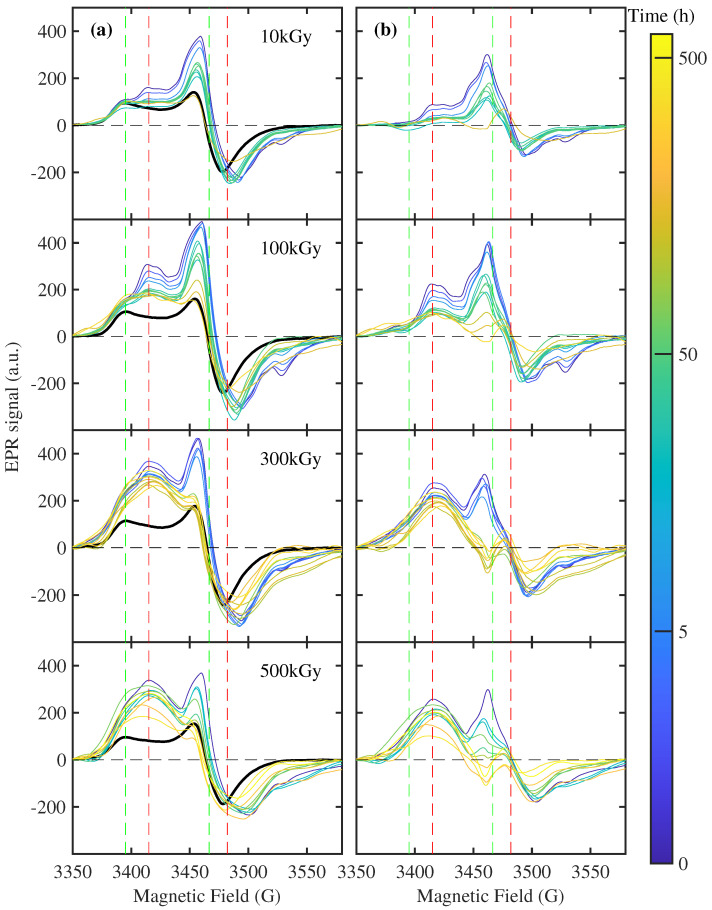
Temporal evolution of EPR spectra of EA. In (**a**), the raw spectral profiles are reported at different doses. The color of each thin line indicates the time delay with respect to the end of gamma irradiation (see the color bar). The EPR spectra before irradiation are reported with black lines. The difference of the EPR spectrum with respect to the nonirradiated sample is reported in (**b**). The green and red vertical dashed lines point to the magnetic field value in correspondence with *g* contributions observed in nonirradiated and irradiated samples, respectively. The samples are sliced from the bulk specimens with a size > 2 mm.

The breaking of epoxy rings generates crosslink effects through the recombination of molecular free radicals. However, γrays can enhance the free radical density or generate new radicals. The detection of such free radicals through EPR analysis represents a powerful method to characterize polymers [33]. The EPR spectrum of EA before gamma irradiation is reported with the black line in Figure 6a. The observed dispersive profile at 3466 G and the peak at 3395 G can be ascribed to the pristine oxygen free radicals of the peroxide group [34], generated by the breaking of the epoxy ring. Specifically, these two features were assigned to the parallel ($g_{\|}$) and perpendicular ($g_{⊥}$) components of the anisotropic *g* tensor, which can be distinguished in the case of long correlation time of molecular motion ($\tau ≳3\times 10^{-8}$ s [34]). In contrast with the case of PTFE [34], in which the components $g_{⊥}$ and $g_{\|}$ can be distinguished only at low temperatures, our samples preserve a slow τ also at room temperature.

As expected, the interaction of γray with EA induces an enhancement of the EPR signal (see blue lines in Figure 6a) due to an increase in free radicals. However, the EPR shape is consistently different with respect to the black lines, suggesting the excitation of a new type of free radicals. For a dose of 10 kGy, a new contribution similar to the nonirradiated sample can be observed in Figure 6b, suggesting a similar atomic source for these free radicals. However, as highlighted by the green and red dashed lines, there is a shift of the spectral features of 15–20 G, corresponding to a reduction in g-value and then a different chemical environment for this type of radical.

**Figure 7:**
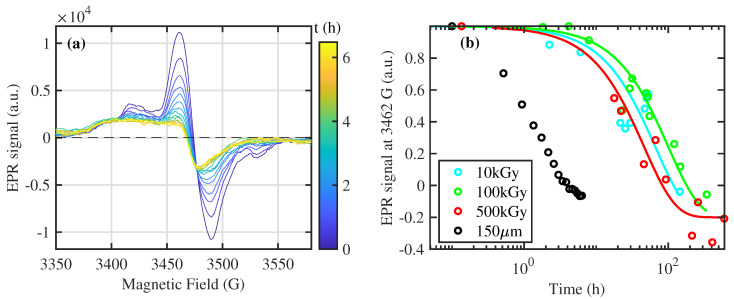
Temporaldynamic of EPR signal in EA. (**a**) The EPR spectra of the EA samples with a thickness of 150 µm are reported. The color of each thin line indicates the time delay with respect to the end of gamma irradiation at 50 kGy (see the color bar). (**b**) The transient EPR intensity at 3462 G (Figure 6b), normalized to 1 for the first measurement after irradiation for each absorbed dose, is reported as a function of the recovery time through colored circles. The black circles represent the time profile for the sample with a thickness of 150 µm, reported in (**a**). The solid lines represent the exponential best fit of the experimental data with time scales of 66, 100, and 64 h for 10, 100, and 500 kGy, respectively.

The temporal recombination of free radicals is strictly related to the chemical environment of EA. In particular, the exposition to air also during the irradiation procedure is crucial. For this reason, the experiment reported in Figure 6 for bulk specimens is repeated in Figure 7a on a sample with a thickness of 150 µm and irradiated at 50 kGy. Here, the average exposition of polymeric chain to the air increases, and, consequently, the recombination time (i.e., the time requested to reduce the signal by a factor *e*) is decreased by more than one order of magnitude, i.e., from ∼70 to ∼2 h. This effect can be explained by a thickness-dependent capability in the diffusion of environment molecules (i.e., oxygen and water), able to recombine the free radicals of EA [35]. However, the time evolution of the EPR spectra is similar to that of the thick samples. This dependence on air exposition during irradiation should be considered for the application of EA in space or nuclear activities.

**Figure 8:**
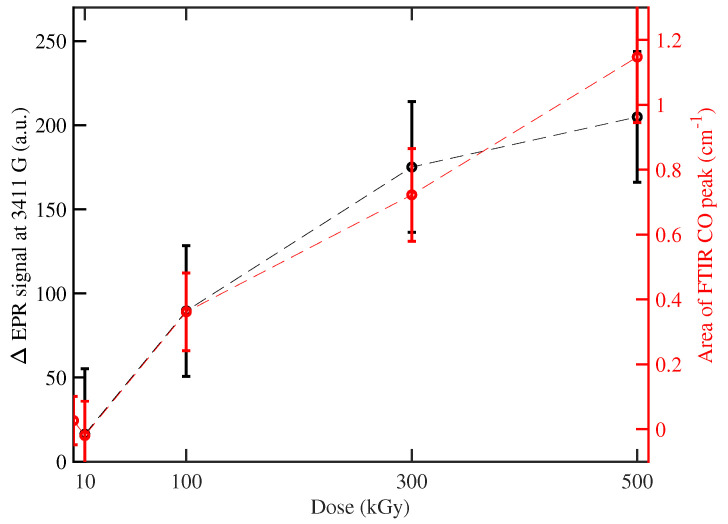
Dose dependence of EPR signal difference (see Figure 6b) at 3411 G and area of C=O peak in FTIR measurement, in the left and right axis, respectively. The area of the C=O peak is calculated between 1700 and 1800 cm^−1^ [20]. The two profiles are compatible considering the error of measurement. Part of the figure was reproduced/adapted with permission from [20], Elsevier, 2021.

In Figure 6, at higher doses, an additional broader EPR contribution at ∼3415 G is gradually superimposed to the previous ones. This large contribution can be ascribed (i) to oxygen-free radicals of the peroxide group with high mobility (in which τ is shorter than the pristine contribution) and (ii) to a different chemical species ionized by gamma radiation. It does not exhibit an evident decrease in the time scale of our measurements and follows the same dose dependence of structural degradation, quantified by the area of C=O peak in the FTIR measurement (see Figure 8). This analogy demonstrates that this type of free radical is the same affected by the C=O recombination observed by FTIR.

Moreover, Figure 6a shows, at the largest dose of 500 kGy, a decrease in the signal at 3462 G below the nonirradiated sample for recovery time > 200 h, indicating a recombination of pristine free radicals also at higher doses, concomitant with the degradation process observed by mechanical characterization [20] and FTIR spectroscopy.

## 4. Conclusions

The results provide a detailed spectroscopic analysis of the complex mechanism induced by the γrays. Their capability to interact not selectively with each chemical component of polymers induces in the material a mix of effects, observed macroscopically by a modification of mechanical properties [20,21]. Our work dissects these multifactorial effects in EA. Specifically, we observe the fragmentation of polymeric chains, demonstrated by the increase in the FTIR peak connected to the C=O complex, the broadening of the FTIR peaks, and the generation of new free radicals, evidenced by EPR measurements. However, simultaneously with the degradation of polymers, there is also an increase in crosslinks, testified by the shift of FTIR peak at 827 cm^−1^ and the recombination of free radicals. These two effects do not affect the response in the THz-TDS regime, demonstrating a negligible modification of the crystallinity of the EA. Moreover, we find analogies in the time evolution of the evidenced structures between EPR and FTIR measurement, testified by the similar dose dependence of EPR peak ∼3411 G and of the FTIR C=O peak at ∼1725 cm^−1^, which is related to the scission of polymeric chains. This analogy indicates a new EPR feature, related to the structural modification of EA.

By studying the EPR signal after gamma irradiation, the temporal evolution of free radicals was extracted and we found a strong dependence on the thickness of the sample, which induces modification of the diffusion of environment molecules in EA. Summing up, these experimental observations allow us to conclude the following:

- The γrays exposure induces several free radicals at low exposure (10 kGy). The dependence on sample thickness demonstrates a recombination mediated by the exposure to the gaseous environment.
- At higher exposure (>100 kGy), the FTIR spectra show a dose-dependent structural modification ascribable to the scission of polymeric chains and the increase in crosslinking between polymeric chains. To explain this effect, we should consider that such structural modification can be realized by the generation of free radicals in the same spatial region [5]. This specific condition can be obtained only by increasing the γrays exposure.

## Data Availability

Data are contained within the article.

## Abbreviations

The following abbreviations are used in this manuscript:

EA	Epoxy adhesive
FTIR	Fourier transform infrared spectroscopy
TDS	Time-domain spectroscopy
EPR	Electron paramagnetic resonance
HEP	High-energy physics
γrays	Gamma rays
PTFE	Polytetrafluoroethylene
PA	Photoconductive antenna
PCA	Principal component analysis
PC1	First principal component
PC2	Second principal component
$g_{\|}$	Parallel components of the anisotropic *g* tensor
$g_{⊥}$	Perpendicular components of the anisotropic *g* tensor
h	hour
